# Impact of frailty on outcomes of inpatient stereotactic radiosurgery for brain metastasis: a national readmission database analysis 2016–2020

**DOI:** 10.1186/s13014-025-02750-4

**Published:** 2025-11-28

**Authors:** Ryan Wing Yuk Chan, Chien-Kai Wang, Wei-Lun Lo, Tu-Hsueh Yeh, Niramol Savaraj, Lynn G. Feun, Shu-Mei Chen

**Affiliations:** 1 Department of Neurosurgery, Taipei Medical University Hospital, Taipei Medical University, Taipei, Taiwan; 2 Ph.D. Program in Medical Neuroscience, College of Medical Science and Technology, Taipei Medical University, Taipei, Taiwan; 3 Department of Surgery, School of Medicine, College of Medicine, Taipei Medical University, Taipei, Taiwan; 4 Taipei Neuroscience Institute, Taipei Medical University, 250 Wu-Hsing Street, Taipei, 110 Taiwan; 5 Department of Neurosurgery, Shuang Ho Hospital, Taipei Medical University, Taipei, Taiwan; 6 Department of Neurology, Taipei Medical University Hospital, Taipei Medical University, Taipei, Taiwan; 7 Department of Neurology, School of Medicine, College of Medicine, Taipei Medical University, Taipei, Taiwan; 8 Department of Hematology/Oncology, Miami VA Healthcare System, Miami, FL USA; 9 Division of Hematology/Oncology, Sylvester Comprehensive Cancer Center, University of Miami Miller School of Medicine, Miami, FL USA

**Keywords:** Frailty, Stereotactic radiosurgery (SRS), Brain metastasis, National readmission database (NRD), Outcomes

## Abstract

**Background:**

It is not clear how frailty may affect the outcomes of stereotactic radiosurgery (SRS) for brain metastasis. This study aimed to evaluate the impact of frailty on clinical outcomes in patients ≥ 60 years old who underwent SRS for brain metastasis from a population-based perspective.

**Materials and methods:**

Data were extracted from the National Readmission Database (NRD), 2016 to 2020. Inclusion criteria were ≥ 60 years old with brain metastasis who underwent SRS. Frailty was assessed using the modified Frailty Index (mFI), derived from 11 clinical conditions. The primary outcomes were in-hospital mortality, length of hospital stay (LOS), total hospital costs, and 30-day and 90-day readmission rates. Logistic and linear regression models were used to assess the association between frailty and outcomes.

**Results:**

A total of 904 patients (mean age: 71 years, 53% male) were included, of which 17.5% were defined as frail. After adjusting for demographic, clinical, and hospital-related factors, frailty was significantly associated with increased in-hospital mortality (adjusted odds ratio [aOR] = 2.39, 95% confidence interval [CI]: 1.16–4.92), longer LOS (adjusted Beta [aBeta] = 2.61 days, 95% CI: 1.95–3.28), higher total costs (aBeta = $36.04 thousand USD, 95% CI: 28.84–43.23), and higher 30-day readmission rate (aOR = 1.47, 95% CI: 1.02–2.11).

**Conclusion:**

Frailty independently predicts poorer outcomes in older adults undergoing SRS for brain metastasis, including higher mortality, longer hospital stays, increased hospital costs, and increased 30-day readmission rate. These findings highlight the importance of incorporating frailty-informed risk stratification and perioperative care planning to optimize patient outcomes.

**Trial registration number:**

Not applicable.

**Supplementary Information:**

The online version contains supplementary material available at 10.1186/s13014-025-02750-4.

## Introduction

Brain metastasis is a common complication in patients with cancer [1]. It is particularly prevalent in patients with advanced lung, breast, and melanoma cancers [2]. Treating brain metastases is challenging due to the brain’s complex nature, and the need to preserve neurological function while effectively controlling tumor growth [3, 4]. Stereotactic radiosurgery (SRS) has emerged as a preferred treatment for brain metastases because of its precision in delivering high doses of radiation directly to the tumor, minimizing damage to surrounding healthy tissues [5]. SRS has revolutionized the management of brain metastases by offering improved local control and, in some cases, enhancing patient survival without the invasiveness of traditional surgery [6, 7].

Frailty is a syndrome characterized by diminished physiological reserves and increased vulnerability to stressors [8]. It is commonly observed in older patients and those with chronic diseases, including cancer [9]. Frail individuals are at higher risk for postoperative complications, longer hospital stays, and decreased overall survival [10, 11]. As the population ages, understanding the interaction between frailty and treatment outcomes has become crucial for optimizing patient care in patients receiving surgeries or any treatment intervention.

To the best of our knowledge, no prior study has examined how frailty influences the outcomes of patients with malignant brain metastases undergoing SRS. By examining this relation using a large national dataset, we seek to provide insights that could guide personalized treatment strategies and optimize outcomes for these patients. Although SRS is typically performed in outpatient settings, some patients require hospitalization due to acute neurological symptoms, significant comorbidities, or closer monitoring needs. This study focuses on these hospitalized patients, who may represent a more complex subgroup.

## Methods

### Data source

The Nationwide Readmissions Database (NRD) is a United States database of all-payer hospital inpatient stays that can be used to generate national estimates of readmissions. The NRD is a 100% sample from HCUP State Inpatient Databases (SID) with discharge- and hospital-level exclusions, containing verified patient linkage numbers that can be used to track a person across hospitals within a State, while adhering to strict privacy guidelines. The NRD contains data for an entire calendar year, with diagnoses and procedures reported using the International Classification of Diseases, Tenth Edition, Clinical Modification (ICD-10-CM), and Procedure Coding System (PCS), starting from the 2016 data year. More details are available at: https://hcup-us.ahrq.gov/nrdoverview.jsp.

### Study design and population

This population-based retrospective study included patients aged 60 years or older who were first admitted to undergo SRS for brain metastasis between January 1 and September 30 of each year from 2016 to 2020. The admission window was restricted to January through September to allow complete 90-day follow-up within the same calendar year, as the NRD does not track patients across years. Patients were excluded if they had undergone craniotomy or had missing data of key variables, such as primary endpoints, sex, or sample weight. Disease identification was based on International Classification of Diseases, 10th Revision (ICD-10) codes. From the time of their index admission, patients were considered at risk for hospitalization, and follow-up data until December 31 of the admission year or death were available. A comprehensive list of the ICD codes used can be found in Supplementary Table S1.

### Ethics statement

All data from NRD were anonymized and devoid of personally identifiable information, ensuring compliance with ethical standards for research. As this research involved the use of secondary data, no patients were directly involved and the requirement of informed consent was waived.

### Determination of frailty status using the modified frailty index

The modified Frailty Index (mFI) was developed using 11 variables derived from the Canadian Study of Health and Aging Frailty Index [12]. The 11 variables are: (1) diabetes mellitus (DM); (2) chronic obstructive pulmonary disease (COPD); (3) congestive heart failure; (4) myocardial infarction; (5) history of percutaneous coronary intervention, cardiac surgery, or angina; (6) hypertension; (7) impaired sensorium; (8) peripheral vascular disease or rest pain; (9) cerebrovascular accident with deficit; (10) cerebrovascular accident without deficit; and (11) dependent functional status. Each of these conditions contributes 1 point to the mFI score. The final score is calculated by dividing the total number of positive variables by 11, resulting in a score that ranges from 0 to 1.0. A score of ≥ 0.27 indicates frailty.

### Study outcomes and variables

Patient characteristics collected for this study included age, sex, and insurance status (primary payer). Specific health conditions assessed were smoking status, obesity, primary tumor type, and current use of antiplatelet drugs, anticoagulants, and systemic steroids. Comorbid conditions associated with brain metastasis were evaluated using the Charlson Comorbidity Index (CCI). Hospital-related factors were also analyzed, including bed size, location, teaching status, and whether the admission occurred on a weekend.

The study outcomes were in-hospital mortality, length of hospital stay (LOS), total hospital costs, 30-day and 90-day readmission rates, and complications. Major complications evaluated were edema, seizures, hydrocephalus, cognitive decline, cranial nerve injury, hearing loss, visual disturbances, radiation-induced leukoencephalopathy, hypopituitarism, dysphagia, and dysphonia. Complications were further categorized by the number of occurrences: 0, 1, and 2 or more.

### Statistical analysis

To generate national estimates, all analyses accounted for the NRD’s complex survey design, incorporating sampling weights, stratification, and clustering. Descriptive statistics for patients who were admitted with a primary or secondary discharge diagnosis of brain metastasis and received SRS at the time of index admission were presented as either the number (n) and weighted percentage (%), or mean and standard error (SE). Categorical variables were compared using the Rao-Scott chi-square test to account for the complex survey design, while continuous variables were compared using survey-weighted t-tests. Logistic regressions were performed to determine the effect of frailty on in-hospital mortality, complications, and readmission rates, and data were reported as odds ratio (OR) and 95% confidence interval (CI). Linear regressions were performed to determine the association between frailty, LOS, and total hospital costs; data were reported as estimate coefficients (Beta) and 95% CI. The covariates with a p-value < 0.1 in univariate analysis were considered relevant factors (except for CCI), and were adjusted for in the multivariable models. All p-values were 2-sided, and p-values of < 0.05 were considered statistically significant. Statistical analyses were performed using SAS software version 9.4 (SAS Institute Inc., Cary, NC, USA).

## Results

### Patient selection

**Fig. 1:**
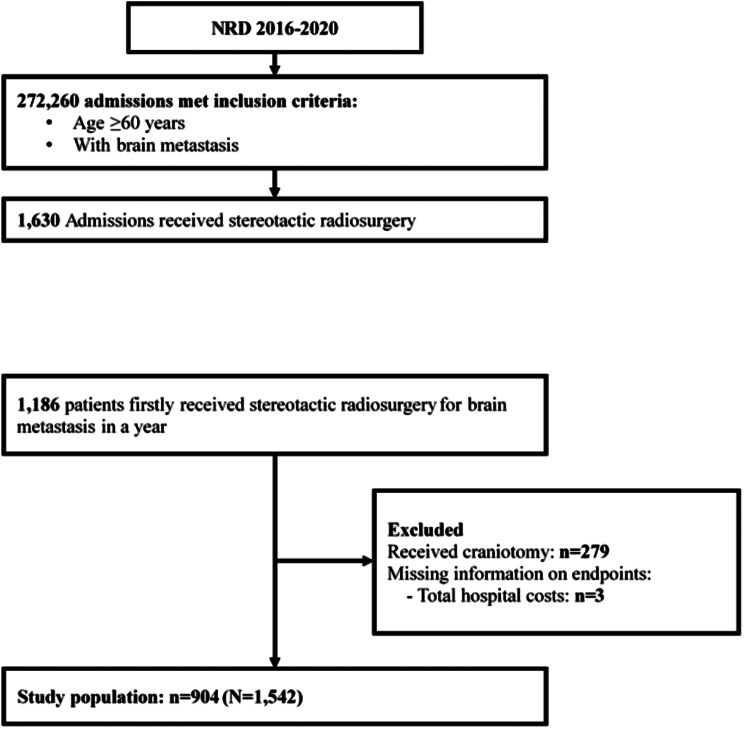
Flow diagram of patient selection

The patient selection process is illustrated in Fig. 1. Review of the NRD database between 2016 and 2020 identified 1,193 patients ≥ 60 years old diagnosed with brain metastasis who received SRS between January 1 and September 30 each year. Of these patients 1,186 had their first SRS treatment, and patients who received craniotomy (*n* = 279) or had missing information on hospital costs (*n* = 3) were excluded. Finally, a total of 904 patients were included in the study, representing a total of 1,542 hospitalizations in the United States.

### Patient characteristics

The mean age of the patients was 71 years and 53% were males. The majority were insured by Medicare/Medicaid (80%), 48% had respiratory cancer, 53% had a CCI of 0–1, 78% were treated at a large hospital, and 87% were treated at a metropolitan teaching hospital. Of the patients, 158 (18%) were defined as frail. Frail patients were older (mean age 73 vs. 71 years), and had higher proportions of male, Medicare/Medicaid insurance coverage, used antiplatelet drugs, used anticoagulants, and had a CCI ≥ 2 (Table 1).

**Table 1:** Characteristics of the study population

Characteristics	Total (*N* = 904)	Frailty		*p*-value
		Yes (*n* = 158)	No (*n* = 746)	
**Demography**				
**Age**,** years**	71.1 ± 0.2	72.5 ± 0.6	70.8 ± 0.3	**0.010**
60–69	416 (46.1)	61 (37.7)	355 (47.8)	0.062
70–79	346 (38.3)	69 (45.1)	277 (36.9)	
80+	142 (15.6)	28 (17.2)	114 (15.3)	
**Sex**				**0.019**
Male	474 (52.7)	95 (60.4)	379 (51.1)	
Female	430 (47.3)	63 (39.6)	367 (48.9)	
**Insurance status / Primary payer**				
Medicare/Medicaid	715 (79.4)	135 (85.6)	580 (78.1)	**0.028**
Private including HMO	154 (16.6)	16 (9.2)	138 (18.1)	
Self-pay/no-charge/other	35 (4.0)	7 (5.2)	28 (3.8)	
**Smoking**	490 (53.5)	82 (50.1)	408 (54.2)	0.366
**Obesity**	98 (11.6)	24 (15.0)	74 (10.9)	0.151
**Primary tumor**				0.422
Gastrointestinal cancers	52 (5.6)	9 (5.8)	43 (5.6)	
Respiratory cancer	434 (47.8)	90 (55.5)	344 (46.2)	
Melanoma	42 (4.6)	6 (3.6)	36 (4.8)	
Breast cancer	44 (4.7)	4 (2.2)	40 (5.2)	
Gynecologic cancer	20 (2.5)	1 (0.9)	19 (2.8)	
Male-specific cancer	14 (1.5)	1 (1.3)	13 (1.5)	
Urinary tract carcinoma	48 (5.4)	9 (5.3)	39 (5.5)	
Others	250 (27.8)	38 (25.3)	212 (28.3)	
**Current use of**:				
Antiplatelet drugs	126 (14.1)	38 (24.4)	88 (11.9)	**< 0.001**
Anticoagulants	70 (8.1)	23 (16.2)	47 (6.4)	**< 0.001**
Systemic steroids	25 (2.8)	5 (3.4)	20 (2.7)	0.649
**CCI**				**< 0.001**
0–1	477 (52.9)	14 (9.3)	463 (62.0)	
2–3	281 (30.9)	75 (47.9)	206 (27.4)	
4+	146 (16.1)	69 (42.8)	77 (10.6)	
**Hospital status**				
**Weekend admission**	216 (23.8)	44 (28.7)	172 (22.8)	0.123
**Hospital bed size**				0.663
Small	39 (4.3)	6 (4.0)	33 (4.4)	
Medium	156 (17.3)	32 (18.9)	124 (17.0)	
Large	709 (78.3)	120 (77.1)	589 (78.6)	
**Hospital location/teaching status**				0.912
Metropolitan non-teaching	99 (10.4)	16 (10.4)	83 (10.4)	
Metropolitan teaching	786 (86.6)	138 (86.2)	648 (86.7)	
Non-metropolitan hospital	19 (3.0)	4 (3.4)	15 (2.9)	

CCI: Charlson Comorbidity index; HMO, Health Maintenance Organization

Significant values are shown in bold

### In-hospital outcomes

In-hospital mortality, complications, LOS, total hospital cost, and 30-day and 90-day readmission rates were compared between frail and non-frail patients. Frail patients had a longer mean LOS (13.4 vs. 10.4 days, *p* < 0.001) and higher total hospital costs ($193.4 vs. $156.7 thousand USD, *p* < 0.001). Overall complication rates were not significantly different between the two groups (*p* = 0.819), but patients with frailty had a higher 30-day readmission rate (34% vs. 26%, *p* = 0.032) (Table 2).

**Table 2:** In-hospital outcomes

Characteristics	Total (*N* = 904)	Frail		*p*-value
		Yes (*n* = 158)	No (*n* = 746)	
Outcome				
**In-hospital mortality**	34 (3.3)	10 (5.6)	24 (2.9)	0.052
**LOS**,** days**^**a**^	10.9 ± 0.3	13.4 ± 0.8	10.4 ± 0.4	**< 0.001**
**Total hospital cost**,** per 1000 USD**^**a**^	162.8 ± 4.4	193.4 ± 11.1	156.7 ± 4.2	**< 0.001**
**Complication**				0.819
0	277 (29.9)	48 (30.8)	229 (29.7)	
1	494 (55.6)	89 (56.3)	405 (55.5)	
2+	133 (14.4)	21 (12.8)	112 (14.8)	
**Neurological complications**				
**Central structural / pressure-related**				
Edema	574 (64.8)	100 (63.3)	474 (65.2)	0.646
Hydrocephalus	38 (3.9)	6 (3.9)	32 (3.9)	0.977
**Central functional manifestations**				
Seizures	28 (2.8)	6 (3.2)	22 (2.7)	0.745
Cognitive decline	3 (0.3)	-	3 (0.4)	-
**Treatment-related**				
Radiation-induced leukoencephalopathy	14 (1.5)	3 (1.9)	11 (1.5)	0.706
**Cranial nerve and sensory complications**				
Cranial nerve injury	2 (0.2)	-	2 (0.2)	-
Hearing loss	21 (2.2)	4 (2.0)	17 (2.2)	0.843
Visual disturbance	34 (3.4)	3 (1.8)	31 (3.7)	0.225
**Endocrine complications**				
Hypopituitarism	3 (0.6)	-	3 (0.7)	-
**Swallowing and speech-related complications**				
Dysphagia	52 (5.6)	11 (7.1)	41 (5.3)	0.295
Dysphonia	2 (0.2)	-	2 (0.2)	-
**30-day readmission rate** ^**a**^	241 (26.9)	51 (33.6)	190 (25.6)	**0.032**
**90-day readmission rate** ^**a**^	414 (46.5)	78 (52.0)	336 (45.3)	0.124

LOS, length of stay

^a^ Excluding patients who died in the hospital

Significant values are shown in bold

### Associations between frailty and in-hospital outcomes

After adjusting for relevant factors, the analysis showed that frailty had a significant impact on in-hospital mortality, LOS, total hospital costs, and 30-day readmission rate. Compared to patients without frailty, those with frailty had increased risks of in-hospital mortality (adjusted odds ratio [aOR] = 2.39, 95% confidence interval [CI]: 1.16, 4.92, *p* = 0.019), 30-day readmission (aOR = 1.47, 95% CI: 1.02, 2.11, *p* = 0.038), longer LOS (adjusted Beta [aBeta] = 2.61, 95% CI: 1.95, 3.28, *p* < 0.001), and higher total hospital costs (aBeta = 36.04, 95% CI: 28.84, 43.23, *p* < 0.001) (Table 3, Supplemental Tables S2 and S3).

**Table 3:** Associations between frailty (frail vs. not frail) and in-hospital outcomes

Outcomes	Univariate		Multivariable	
	OR (95% CI)	*p*-value	aOR (95% CI)	*p*-value
**In-hospital mortality** ^**a**^	2.00 (0.98, 4.07)	0.056	2.39 (1.16, 4.92)	**0.019**
**Complication** ^**b**^	0.95 (0.66, 1.37)	0.783	0.93 (0.64, 1.35)	0.697
**30-day readmission rate** ^**c**^	1.47 (1.03, 2.11)	**0.035**	1.47 (1.02, 2.11)	**0.038**
	**Beta (95% CI)**	**p-value**	**aBeta (95% CI)**	**p-value**
**LOS**,** day**^**d**^	3.05 (2.37, 3.74)	**< 0.001**	2.61 (1.95, 3.28)	**< 0.001**
**Total hospital cost**,** per 1000 USD**^**e**^	36.69 (28.64, 44.73)	**< 0.001**	36.04 (28.84, 43.23)	**< 0.001**

LOS, length of stay; CI, confidence interval; OR, odds ratio; aOR, adjusted OR; aBeta, adjusted Beta

All outcomes excluded patients who died in the hospital: except the outcomes of in-hospital mortality and complications

^a^ Adjusted for age, primary tumor, hospital bed size

^b^ Adjusted for smoking status, weekend admission, hospital bed size, and hospital location/teaching status

^c^ Adjusted for hospital bed size

^d^ Adjusted for sex, insurance status, smoking, obesity, primary tumor, antiplatelets, systemic steroid, hospital bed size, hospital location/teaching status

^e^ Adjusted for age, sex, smoking, primary tumor, antiplatelet drug use, anticoagulants, systemic steroids, hospital bed size, hospital location/teaching status

### Sensitivity analysis

In addition, we performed sensitivity analyses using alternative frailty specifications and stratification by hospital type, which showed consistent results and further support the robustness of our findings (Supplemental Table S4).

## Discussion

This study revealed that frailty is a significant predictor of adverse outcomes in hospitalized patients ≥ 60 years who were undergoing SRS for brain metastasis. Frail patients had notably higher in-hospital mortality, longer LOS, increased hospital costs, and a significantly higher 30-day readmission rate compared to non-frail patients. Specifically, frailty was associated with a 2.4-fold increase in the risk of in-hospital mortality, a mean LOS increase of about 3 days, and approximately $36,040 in additional hospital costs. These findings underscore the critical impact of frailty on clinical outcomes, highlighting the need for frailty-informed risk stratification and enhanced perioperative care in this population. From a clinical and health system perspective, these effect sizes are substantial. An additional 3 days of hospitalization not only increases direct medical costs but also consumes high-acuity neurosurgical or oncology beds, which are often limited resources in tertiary centers. This prolonged stay may delay care for other patients requiring urgent interventions. Likewise, an aOR of 2.4 for in-hospital mortality means that frail patients have more than double the risk of death during the index admission—a magnitude of risk that is highly relevant for patient counseling and perioperative planning. For hospital administrators, these results emphasize the value of early frailty screening, proactive optimization before SRS, and targeted discharge planning to reduce mortality, readmissions, and resource strain. Integrating frailty assessment into routine workflows can guide multidisciplinary discussions, inform allocation of specialized nursing or rehabilitation services, and support value-based care initiatives.

As previously mentioned, we believe this is the first study to examine the impact of frailty on the outcomes of patients with brain metastases undergoing SRS. However, a number of studies have examined the influence of frailty on patients with primary brain tumors and metastases undergoing resection [13–18]. For example, a study of 260 patients undergoing surgery for brain tumor resection reported that frailty was significantly associated with discharge to a location other than home, postoperative complications, and longer LOS [18]. An analysis of the records of about 11,000 patients who underwent resection of brain metastases reported that frailty calculated using the Risk Analysis Index was predictive of 30-day mortality [16]. Kerschbaumer et al. ^17^ also studied frailty in patients with brain metastases undergoing surgical resection, and reported frailty calculated using the Clinical Frailty Scale was predictive of overall survival (OS) following surgery. A recent systematic review also found that indices of frailty were useful predictors of outcomes in patients undergoing surgical resection of brain metastases; however, the authors cautioned that further validation of the indices is warranted [13].

Assessment of frailty is being used in many medical and surgical disciplines to help stratify patient risk and predict outcomes such that management may be altered in order to provide a higher level of care in patients at higher risk of adverse outcomes. Studies are beginning to assess the value of frailty assessment in radiation oncology, and especially in geriatric radiation oncology, and authors’ have recommended a Comprehensive Geriatric Assessment (CGA) with the use of frailty screening tools to assist in the decision-making process for older patients who require radiation treatment for malignancies [19]. The Geriatric-8 score is one of the most validated measure of frailty in geriatric patients, and is being studied in geriatric patients undergoing radiation therapy [20]. Fernández-Camacho et al. [21] recently reported that radiation oncologists modified radiation treatment plans based on frailty identified using a CGA and the Geriatric-8 score. In a study of men with prostate cancer treated with radiotherapy, Pan et al. [22] reported that severe frailty assessed using the mFI was associated with a higher risk of all-cause mortality and cancer-specific mortality.

Surprisingly, our results showed that frail patients were not at an increased risk of complications during hospitalization. While this may seem counterintuitive given their reduced physiological reserve, one possible explanation is the precision of stereotactic radiosurgery (SRS), which minimizes damage to surrounding healthy tissue. Unlike traditional surgeries that may impose greater systemic stress, SRS may be better tolerated by frail patients with respect to the complications evaluated in our analysis. Another potential explanation is that, since this study relies on an administrative database based on coding and lacks clinical variables such as laboratory parameters, subtle clinical changes may have been underreported, and minor or detailed complications could have been missed, introducing potential bias.

The elevated mortality and readmission rates in frail patients might be linked to the cumulative effects of underlying comorbidities and decreased physiological reserves. A recent study by Shinall et al. [23] reported that mortality rates for frail patients undergoing low- and moderate-stress surgical procedures were significantly higher than for non-frail patients. The authors thus concluded that treatments considered low-risk for healthy patients may pose significant risks for frail patients. Similarly, in a review by Bhattarai et al. [24] discussed the relation between frailty, which is decline in reserve capacity across multiple organ systems, and multimorbidity (the presence of 2 or more chronic diseases). Frail persons are more likely to develop chronic diseases, and persons with chronic diseases are more likely to become frail. Because frail individuals often have a higher burden of chronic illnesses (e.g., cardiovascular disease, respiratory conditions), the stress associated with radiation treatment may make SRS “high-risk” even though the treatment itself is minimally invasive.

Our results also showed that patients treated at large hospitals had a 67% lower risk of in-hospital mortality (Supplementary Table S1). This outcome is not unexpected as many studies have showed that hospitals that perform a large volume of specific procedures and treatments (and are usually large hospitals) overall have better outcomes than those at hospitals where the volume of the procedure or treatment is relatively low. McClelland et al. [25] reported that the survival of patients with brain metastases treated with radiation therapy is significantly higher at hospitals that perform 12 or more procedures per year as compared to lower volume hospitals.

With the aging population, the numbers of elderly and very old persons with brain tumors or metastases will increase, and despite the effect of frailty on outcomes, SRS remains one of the most effective treatments, offering significantly fewer complications compared to open surgery and other modalities [26]. Although SRS is typically performed as an outpatient procedure, a subset of patients requires inpatient admission due to severe neurological symptoms, significant medical comorbidities, or the need for closer perioperative monitoring. Accordingly, our study population represents hospitalized patients undergoing SRS, who likely have a higher burden of comorbidities and greater clinical complexity compared to the broader outpatient SRS population. These factors should be considered when interpreting the generalizability of our findings.

Taken together, our findings and those of other studies underscore the importance of assessing as well as treating frailty before medical and surgical treatments. This may include prehabilitation programs designed to improve the physical and nutritional status of frail patients before SRS, more frequent post-treatment follow-ups to catch complications early, and comprehensive management of underlying comorbidities. Additionally, personalized dose adjustment or multi-disciplinary collaboration between oncologists, geriatricians, and palliative care specialists could help optimize outcomes for frail patients.

### Strengths and limitations

This study’s strengths include its large, nationally representative sample drawn from the NRD, which allows for generalizability of the findings across a wide population of older adults undergoing SRS for brain metastasis. The use of a validated mFI, derived from established clinical variables, provides a reliable method to quantify frailty and assess its impact on surgical outcomes. The mFI is widely accepted in clinical research and has been shown to predict outcomes in various surgical populations, enhancing the robustness of the study’s conclusions.

However, the study also has several limitations. One key limitation is its reliance on administrative data, which inherently lacks granular clinical information. For instance, the dataset does not provide detailed information on tumor characteristics, such as size, location, or histology, which could influence treatment outcomes. In addition, the SRS procedure codes used do not distinguish between treatment platforms, lesion number, or dosing schemes. These factors could affect patient outcomes and their omission may limit the ability to fully account for all variables influencing prognosis. Second, the retrospective nature of the study introduces potential selection bias, as patients deemed too frail might not have been offered SRS at all, potentially underestimating the true impact of frailty on outcomes. Third, while the frailty index used in this study focuses on comorbidities, it may not fully capture other critical dimensions of frailty, such as functional impairment, nutritional status, and cognitive decline, which are important predictors of surgical outcomes in older adults. Frailty is a multifaceted syndrome, and a more comprehensive assessment—including functional and cognitive measures—might provide a more accurate prediction of postoperative risk. Fourth, the NRD database does not contain information on the specific causes of in-hospital death. Therefore, it is not possible to distinguish whether mortality resulted from progression of brain metastases, systemic complications, treatment-related factors, or comorbid conditions. Regrettably, it is difficult to define these dynamic clinical details using NRD dataset, given its reliance on administrative and billing codes. Complication data were derived from general ICD codes and not specific to radiation effects. Their low frequency also limited statistical power. Readmission reasons were unavailable in the dataset, preventing further interpretation of clinical context. Fifth, as with all administrative datasets, potential coding errors and misclassification (including SRS procedures) may exist. In addition, cost estimates were standardized using hospital-specific cost-to-charge ratios but were not adjusted for regional wage or inflation differences. Sixth, it is important to acknowledge that SRS for brain metastases is often intended to improve or preserve quality of life rather than solely to prolong survival, particularly in patients with limited life expectancy. Given that frail patients inherently have a poorer baseline prognosis, assessment of quality of life outcomes would provide critical complementary information. However, the NRD database lacks detailed clinical data on functional status, symptom burden, or health-related quality of life measures. Future studies incorporating prospective quality of life assessments in frail patients undergoing SRS would be valuable to fully capture the impact of treatment. Despite these limitations, the study provides real-world information into the potential impact of frailty in the setting of SRS.

## Conclusion

Frailty is a significant predictor of adverse outcomes in older patients undergoing SRS for brain metastasis, associated with higher mortality, prolonged hospital stays, increased costs, and higher readmission rates. Incorporating frailty assessment into clinical decision-making can help identify high-risk patients who may benefit from more intensive perioperative management or alternative treatment approaches.

## Supplementary Information

Below is the link to the electronic supplementary material.

Supplementary Material 1

## Data Availability

Research data are stored in an institutional repository and will be shared upon request to the corresponding author.
