# Supplementary material for: Impact of frailty on outcomes of inpatient stereotactic radiosurgery for brain metastasis: a national readmission database analysis 2016–2020
Source: Radiat Oncol. 2025 Nov 28;21:1. doi: 10.1186/s13014-025-02750-4 (PMC12763895; doi:10.1186/s13014-025-02750-4)
Supplement: Supplementary file 1 — Supplementary Material 1 [file 13014_2025_2750_MOESM1_ESM.docx]

| **Supplemental Table S1. The ICD-10-CM or ICD-10-PCS codes used in the study** | |
| --- | --- |
| **Variable** | **ICD-10** |
| **Inclusion/exclusion** |  |
| Brain metastasis | C79.31 |
| Stereotactic radiosurgery | **ICD-10-PCS:** D020DZZ, D020HZZ, D020JZZ |
| Craniotomy | **ICD-10-PCS:** 005x0ZZ (x=0, 1, 2, 6, 7, 8, 9, A, B, C, D),  005x3ZZ (x=0, 1, 2, 6, 7, 8, 9, A, B, C, D), 005x4ZZ (x=0, 1, 2, 6, 7, 8, 9, A, B, C, D), 008x0ZZ (x=0, 7, 8) 008x3ZZ (x=0, 7, 8) 008x4ZZ (x=0, 7, 8) 00Bx0ZZ (x=0, 1, 2, 6, 7, 8, 9, A, B, C, D), 00Bx3ZZ (x=0, 1, 2, 6, 7, 8, 9, A, B, C, D), 00Bx4ZZ (x=0, 1, 2, 6, 7, 8, 9, A, B, C, D), 00Cx0ZZ (x=0, 1, 2, 3, 4, 5, 6, 7, 8, 9, A, B, C, D) 00Cx3ZZ (x=0, 1, 2, 3, 4, 5, 6, 7, 8, 9, A, B, C, D) 00Cx4ZZ (x=0, 1, 2, 3, 4, 5, 6, 7, 8, 9, A, B, C, D) 00Qx0ZZ (x=0, 1, 2, 6, 7, 8, 9, A, B, C, D) 00Qx3ZZ (x=0, 1, 2, 6, 7, 8, 9, A, B, C, D) 00Qx4ZZ (x=0, 1, 2, 6, 7, 8, 9, A, B, C, D) 00T7xZZ (x=0, 3, 4). |
| **11-item Modified Frailty Index (mFI-11)** |  |
| Diabetes | E10.x, E11.x, Z79.4, Z79.84, Z79.85 |
| COPD/pneumonia | J44, J13-J18 |
| Congestive heart disease | I50 |
| Myocardial infraction | I21 |
| Percutaneous coronary stent/intervention/angina | Z95.5 |
| Hypertension | I10, I11 |
| Impaired sensorium | R41.8x |
| Cerebrovascular accident with neurological damage | I60-I69 with R47 |
| Cerebrovascular accident without neurological damage | I60-I69 without R47 |
| Peripheral vascular disease/rest pain | I73 |
| Dependent functional status | Z73.6 |
| **Complication** |  |
| Edema | G93.6 |
| Seizures | G40.9 |
| Hydrocephalus | G91 |
| Cognitive decline | F09 |
| Cranial nerve injury | H46, H47, G50, G52, S04 |
| Hearing loss | H91 |
| Visual disturbance | H53 |
| Radiation-induced leukoencephalopathy | G93.49 |
| Hypopituitarism | E23.0 |
| Dysphagia | R13.1, R47.02, D50.1 |
| Dysphonia | R49.0 |
| **Covariate** |  |
| Primary tumor | C00-C96 |
| Gastrointestinal cancers | C15-C26 |
| Respiratory cancer | C30-C39 |
| Melanoma | C43-C44 |
| Breast cancer | C50 |
| Gynecologic cancer | C51-C58 |
| Male specific cancer | C60-C63 |
| Urinary tract carcinoma | C64-C68 |
| Others |  |
| Current use of: |  |
| Antiplatelet drugs | Z79.02, Z79.82 |
| Anticoagulants | Z79.01 |
| Systemic steroids | Z79.52 |

ICD, International Classification of Disease; CM, Clinical Modification; PCS, Procedure Code System.

| **Supplemental Table S2. Associations between frailty, in-hospital mortality, complication, and readmission rate** | | | | | | | | | | | | | | | | | |
| --- | --- | --- | --- | --- | --- | --- | --- | --- | --- | --- | --- | --- | --- | --- | --- | --- | --- |
| **Variables** | **In-hospital mortality** | | | | |  | **Complications** | | | | |  | **30-day readmission rate ^a^** | | | | |
|  | **Univariate** | |  | **Multivariable** | |  | **Univariate** | |  | **Multivariable** | |  | **Univariate** | |  | **Multivariable** | |
|  | **OR (95% CI)** | **p** |  | **aOR (95% CI)** | **p** |  | **OR (95% CI)** | **p** |  | **aOR (95% CI)** | **p** |  | **OR (95% CI)** | **p** |  | **aOR (95% CI)** | **p** |
| **Frailty** | 2.00 (0.98, 4.07) | 0.056 |  | 2.39 (1.16, 4.92) | **0.019** |  | 0.95 (0.66, 1.37) | 0.783 | 0 | 0.93 (0.64, 1.35) | 0.697 |  | 1.47 (1.03, 2.11) | **0.035** |  | 1.47 (1.02, 2.11) | **0.038** |
| **Demography** |  |  |  |  |  |  |  |  |  |  |  |  |  |  |  |  |  |
| **Age, year** |  | **0.041** |  |  |  |  |  |  |  |  |  |  |  | 0.533 |  |  |  |
| 60-69 | ref |  |  | ref |  |  | ref |  |  |  |  |  | ref |  |  |  |  |
| 70-79 | 0.43 (0.20, 0.93) | **0.032** |  | 0.42 (0.19, 0.95) | **0.036** |  | 0.85 (0.63, 1.15) | 0.289 |  |  |  |  | 0.95 (0.68, 1.34) | 0.775 |  |  |  |
| 80+ | 0.39 (0.14, 1.14) | 0.087 |  | 0.35 (0.12, 1.02) | 0.055 |  | 1.00 (0.69, 1.44) | 0.986 |  |  |  |  | 1.21 (0.79, 1.84) | 0.376 |  |  |  |
| **Sex** |  |  |  |  |  |  |  |  |  |  |  |  |  |  |  |  |  |
| Male | 1.02 (0.51, 2.02) | 0.966 |  |  |  |  | 1.03 (0.79, 1.34) | 0.815 |  |  |  |  | 1.13 (0.83, 1.54) | 0.449 |  |  |  |
| Female | ref |  |  |  |  |  | ref |  |  |  |  |  | ref |  |  |  |  |
| **Insurance status / Primary Payer** | | 0.332 |  |  |  |  |  |  |  |  |  |  |  | 0.285 |  |  |  |
| Medicare/Medicaid | ref |  |  |  |  |  | ref |  |  |  |  |  | ref |  |  |  |  |
| Private including HMO | 1.70 (0.81, 3.55) | 0.160 |  |  |  |  | 0.90 (0.64, 1.26) | 0.53 |  |  |  |  | 1.36 (0.90, 2.04) | 0.141 |  |  |  |
| Self-pay/no-charge/other | 1.65 (0.37, 7.37) | 0.512 |  |  |  |  | 1.29 (0.58, 2.86) | 0.536 |  |  |  |  | 0.85 (0.34, 2.12) | 0.729 |  |  |  |
| **Smoking** | 0.77 (0.39, 1.52) | 0.449 |  |  |  |  | 1.29 (0.96, 1.72) | 0.089 |  | 1.28 (0.96, 1.72) | 0.093 |  | 1.22 (0.91, 1.63) | 0.186 |  |  |  |
| **Obesity** | 0.74 (0.24, 2.27) | 0.597 |  |  |  |  | 1.34 (0.82, 2.20) | 0.247 |  |  |  |  | 1.23 (0.74, 2.02) | 0.425 |  |  |  |
| **Clinical status** |  |  |  |  |  |  |  |  |  |  |  |  |  |  |  |  |  |
| **Primary tumor** |  | **0.005** |  |  |  |  |  |  |  |  |  |  |  | 0.134 |  |  |  |
| Gastrointestinal cancers | 1.93 (0.56, 6.62) | 0.296 |  | 1.76 (0.50, 6.14) | 0.375 |  | ref |  |  |  |  |  | 2.69 (1.01, 7.18) | **0.048** |  |  |  |
| Respiratory cancer | 1.51 (0.79, 2.86) | 0.209 |  | 1.46 (0.76, 2.80) | 0.255 |  | 1.07 (0.45, 2.58) | 0.878 |  |  |  |  | 2.40 (1.09, 5.26) | **0.029** |  |  |  |
| Melanoma | 2.80 (0.63, 12.45) | 0.176 |  | 3.37 (0.71, 16.11) | 0.127 |  | 0.95 (0.46, 1.96) | 0.89 |  |  |  |  | 1.93 (0.68, 5.47) | 0.217 |  |  |  |
| Breast cancer | ref |  |  | ref |  |  | 1.29 (0.44, 3.80) | 0.64 |  |  |  |  | ref |  |  |  |  |
| Gynecologic cancer | 2.01 (1.26, 3.21) | **0.003** |  | 2.12 (1.23, 3.67) | **0.007** |  | 0.78 (0.27, 2.28) | 0.645 |  |  |  |  | 0.52 (0.10, 2.78) | 0.442 |  |  |  |
| Male-specific cancer ^b^ |  |  |  |  |  |  | 0.52 (0.15, 1.83) | 0.31 |  |  |  |  | 1.86 (0.45, 7.68) | 0.390 |  |  |  |
| Urinary tract carcinoma ^b^ | 2.69 (1.55, 4.67) | **<0.001** |  | 2.72 (1.52, 4.86) | **<0.001** |  | 1.22 (0.46, 3.24) | 0.687 |  |  |  |  | 1.91 (0.78, 4.69) | 0.155 |  |  |  |
| Others |  |  |  |  |  |  | 1.06 (0.50, 2.26) | 0.879 |  |  |  |  | 1.65 (0.75, 3.65) | 0.211 |  |  |  |
| **Current use of:** |  |  |  |  |  |  |  |  |  |  |  |  |  |  |  |  |  |
| Antiplatelet drugs | 0.35 (0.08, 1.46) | 0.149 |  |  |  |  | 1.29 (0.85, 1.97) | 0.229 |  |  |  |  | 1.27 (0.85, 1.91) | 0.242 |  |  |  |
| Anticoagulants | 0.79 (0.18, 3.49) | 0.758 |  |  |  |  | 0.77 (0.46, 1.30) | 0.326 |  |  |  |  | 1.33 (0.78, 2.26) | 0.299 |  |  |  |
| Systemic steroids | NA | **-** |  |  |  |  | 0.96 (0.47, 1.96) | 0.912 |  |  |  |  | 1.05 (0.46, 2.35) | 0.915 |  |  |  |
| **CCI** |  | 0.747 |  |  |  |  |  |  |  |  |  |  |  | **0.001** |  |  |  |
| 0-1 | ref |  |  |  |  |  | ref |  |  |  |  |  | ref |  |  |  |  |
| 2-3 | 1.08 (0.51, 2.33) | 0.835 |  |  |  |  | 1.19 (0.88, 1.61) | 0.259 |  |  |  |  | 1.14 (0.78, 1.67) | 0.490 |  |  |  |
| 4+ | 1.42 (0.58, 3.50) | 0.446 |  |  |  |  | 2.62 (1.70, 4.03) | **<0.001** |  |  |  |  | 2.02 (1.38, 2.96) | **<0.001** |  |  |  |
| **Hospital status** |  |  |  |  |  |  |  |  |  |  |  |  |  |  |  |  |  |
| **Weekend admission** | 1.07 (0.50, 2.29) | 0.857 |  |  |  |  | 1.40 (0.99, 1.99) | 0.057 |  | 1.46 (1.02, 2.09) | **0.039** |  | 1.04 (0.74, 1.47) | 0.808 |  |  |  |
| **Hospital bed size** |  | **0.003** |  |  |  |  |  |  |  |  |  |  |  | **0.021** |  |  |  |
| Small | ref |  |  | ref |  |  | ref |  |  |  |  |  | ref |  |  | ref |  |
| Medium | 0.25 (0.08, 0.76) | **0.015** |  | 0.25 (0.08, 0.81) | **0.021** |  | 1.86 (1.09, 3.16) | **0.022** |  | 1.85 (1.10, 3.11) | **0.020** |  | 1.88 (1.17, 3.02) | **0.009** |  | 1.86 (1.17, 2.95) | **0.008** |
| Large | 0.33 (0.17, 0.65) | **0.001** |  | 0.34 (0.16, 0.75) | **0.007** |  | 1.25 (0.86, 1.81) | 0.247 |  | 1.19 (0.83, 1.71) | 0.348 |  | 1.68 (1.12, 2.51) | **0.012** |  | 1.67 (1.14, 2.45) | **0.009** |
| **Hospital location/teaching status** | | 0.256 |  |  |  |  |  |  |  |  |  |  |  | 0.560 |  |  |  |
| Metropolitan teaching | ref |  |  |  |  |  | ref |  |  |  |  |  | ref |  |  |  |  |
| Metropolitan non-teaching | 1.22 (0.52, 2.83) | 0.644 |  |  |  |  | 0.98 (0.61, 1.58) | 0.948 |  | 1.02 (0.63, 1.67) | 0.925 |  | 1.04 (0.68, 1.60) | 0.849 |  |  |  |
| Non-metropolitan hospital ^b^ |  |  |  |  |  |  | 1.67 (1.12, 2.49) | **0.012** |  | 1.87 (1.20, 2.91) | **0.005** |  | 0.61 (0.25, 1.54) | 0.296 |  |  |  |

CCI, Charlson Comorbidity index; HMO, Health Maintenance Organization; OR, odds ratio; aOR, adjusted OR; CI, confidence interval.

^a^ Excluded patients who died in the hospital.

Significant values are shown in bold.

Covariates with an overall p < 0.1 in the univariate analysis were adjusted in the multivariate models, except for CCI.

^b^ No event occurred in the subgroup. To avoid generating empty set in the logistic model, the following adjustments were made in the univariate and multivariable analysis for in-hospital mortality: (1) Primary tumor: Gynecologic cancer was combined with male-specific cancer; Urinary tract carcinoma was combined with other; (2) Hospital location/teaching status: Non-metropolitan hospitals were combined with metropolitan non-teaching hospitals; (3) NA means no event occurred in the subgroup, therefore, systemic steroid use was not included in the adjustment.

| **Supplemental Table S3. Associations between frailty, length of stay and total hospital costs ^a^** | | | | | | | | | | | |
| --- | --- | --- | --- | --- | --- | --- | --- | --- | --- | --- | --- |
| **Variables** | **Length of stay, day** | | | | |  | **Total hospital costs (per 1000 dollars)** | | | | |
|  | **Univariate** | |  | **Multivariable** | |  | **Univariate** | |  | **Multivariable** | |
|  | **Beta (95% CI)** | **p** |  | **aBeta (95% CI)** | **p** |  | **Beta (95% CI)** | **p** |  | **aBeta (95% CI)** | **p** |
| **Frailty** | 3.05 (2.37, 3.74) | **<0.001** |  | 2.61 (1.95, 3.28) | **<0.001** |  | 36.69 (28.64, 44.73) | **<0.001** |  | 36.04 (28.84, 43.23) | **<0.001** |
| **Demography** |  |  |  |  |  |  |  |  |  |  |  |
| **Age, years** |  | 0.546 |  |  |  |  |  | 0.454 |  |  |  |
| 60-69 | ref |  |  |  |  |  | ref |  |  | ref |  |
| 70-79 | 0.27 (-0.46, 1.00) | 0.461 |  |  |  |  | 0.42 (-7.02, 7.87) | 0.911 |  | -7.09 (-14.37, 0.18) | 0.056 |
| 80+ | -0.12 (-0.95, 0.72) | 0.783 |  |  |  |  | -18.62 (-24.53, -12.70) | **<0.001** |  | -19.43 (-25.67, -13.19) | **<0.001** |
| **Sex** |  |  |  |  |  |  |  |  |  |  |  |
| Male | 1.42 (0.73, 2.10) | **<0.001** |  | 1.28 (0.73, 1.83) | **<0.001** |  | 13.11 (6.98, 19.25) | **<0.001** |  | 17.03 (10.21, 23.84) | **<0.001** |
| Female | ref |  |  |  |  |  | ref |  |  |  |  |
| **Insurance status / Primary Payer** | | **<0.001** |  |  |  |  |  | 0.454 |  |  |  |
| Medicare/Medicaid | ref |  |  |  |  |  | ref |  |  |  |  |
| Private including HMO | -1.33 (-1.83, -0.84) | **<0.001** |  | -1.21 (-1.67, -0.75) | **<0.001** |  | -1.65 (-7.52, 4.22) | 0.581 |  |  |  |
| Self-pay/no-charge/other | 4.49 (3.92, 5.06) | **<0.001** |  | 4.77 (4.05, 5.48) | **<0.001** |  | -4.06 (-10.47, 2.36) | 0.214 |  |  |  |
| **Smoking** | -1.07 (-1.59, -0.55) | **<0.001** |  | -1.15 (-1.60, -0.70) | **<0.001** |  | -8.89 (-16.38, -1.41) | **0.020** |  | -10.18 (-17.23, -3.12) | **0.005** |
| **Obesity** | 2.69 (1.87, 3.51) | **<0.001** |  | 2.33 (1.52, 3.13) | **<0.001** |  | 4.95 (-3.51, 13.42) | 0.251 |  |  |  |
| **Clinical status** |  |  |  |  |  |  |  |  |  |  |  |
| **Primary tumor** |  | **0.001** |  |  |  |  |  |  |  |  |  |
| Gastrointestinal cancers | 0.97 (0.08, 1.86) | **0.032** |  | 0.06 (-0.77, 0.89) | 0.888 |  | 14.05 (3.12, 24.97) | 0.012 |  | 4.87 (-3.20, 12.94) | 0.236 |
| Respiratory cancer | 0.18 (-0.52, 0.88) | 0.606 |  | -0.48 (-1.12, 0.16) | 0.144 |  | 12.90 (2.60, 23.21) | 0.014 |  | 6.21 (-0.69, 13.12) | 0.077 |
| Melanoma | 0.91 (-0.42, 2.24) | 0.180 |  | -0.88 (-2.28, 0.51) | 0.214 |  | 16.33 (0.84, 31.82) | 0.039 |  | -0.35 (-15.27, 14.57) | 0.963 |
| Breast cancer | ref |  |  | ref |  |  | ref |  |  | ref |  |
| Gynecologic cancer | 1.98 (-2.91, 6.88) | 0.426 |  | 1.93 (-2.92, 6.78) | 0.434 |  | 9.36 (-21.84, 40.57) | 0.555 |  | 8.53 (-26.66, 43.72) | 0.634 |
| Male-specific cancer | 4.03 (-0.08, 8.14) | 0.055 |  | 2.67 (-1.41, 6.75) | 0.200 |  | 30.18 (3.87, 56.49) | **0.025** |  | 10.86 (-16.94, 38.66) | 0.443 |
| Urinary tract carcinoma | 0.92 (-0.02, 1.86) | 0.054 |  | -0.23 (-1.23, 0.77) | 0.656 |  | -2.94 (-16.49, 10.62) | 0.670 |  | -14.45 (-27.88, -1.02) | **0.035** |
| Others | -0.28 (-1.16, 0.59) | 0.525 |  | -0.90 (-1.81, 0.00) | 0.050 |  | 10.81 (1.23, 20.40) | **0.027** |  | 4.96 (-3.05, 12.98) | 0.224 |
| **Current use of:** |  |  |  |  |  |  |  |  |  |  |  |
| Antiplatelet drugs | 0.96 (0.27, 1.66) | **0.007** |  | 0.68 (-0.05, 1.41) | 0.067 |  | -7.64 (-15.86, 0.59) | 0.069 |  | -5.85 (-13.26, 1.56) | 0.122 |
| Anticoagulants | 0.52 (-0.32, 1.37) | 0.222 |  |  |  |  | 17.54 (4.46, 30.61) | **0.009** |  | 12.81 (-2.04, 27.66) | 0.091 |
| Systemic steroids | 1.15 (0.42, 1.88) | **0.002** |  | 1.19 (0.43, 1.96) | **0.002** |  | -10.23 (-17.47, -2.98) | **0.006** |  | -14.38 (-20.59, -8.17) | **<0.001** |
| **CCI** |  | **<0.001** |  |  |  |  |  | **<0.001** |  |  |  |
| 0-1 | ref |  |  |  |  |  | ref |  |  |  |  |
| 2-3 | 2.89 (2.35, 3.42) | **<0.001** |  |  |  |  | 23.56 (17.69, 29.44) | **<0.001** |  |  |  |
| 4+ | 4.56 (4.03, 5.10) | **<0.001** |  |  |  |  | 36.35 (30.30, 42.41) | **<0.001** |  |  |  |
| **Hospital status** |  |  |  |  |  |  |  |  |  |  |  |
| **Weekend admission** | -0.40 (-1.03, 0.22) | 0.203 |  |  |  |  | 5.82 (-1.14, 12.78) | 0.101 |  |  |  |
| **Hospital bed size** |  | **<0.001** |  |  |  |  |  | **<0.001** |  |  |  |
| Small | ref |  |  | ref |  |  | ref |  |  | ref |  |
| Medium | -0.18 (-0.58, 0.23) | 0.393 |  | -0.41 (-0.92, 0.11) | 0.122 |  | 31.09 (27.05, 35.14) | **<0.001** |  | 29.57 (24.36, 34.79) | **<0.001** |
| Large | 1.47 (1.04, 1.89) | **<0.001** |  | 1.49 (1.13, 1.85) | **<0.001** |  | 83.47 (79.87, 87.08) | **<0.001** |  | 86.23 (81.96, 90.51) | **<0.001** |
| **Hospital location/teaching status** | | 0.066 |  |  |  |  |  | **<0.001** |  |  |  |
| Metropolitan teaching | ref |  |  | ref |  |  | ref |  |  | ref |  |
| Metropolitan non-teaching | 0.17 (-0.35, 0.68) | 0.532 |  | -0.04 (-0.75, 0.67) | 0.914 |  | -7.45 (-15.82, 0.92) | 0.081 |  | -13.75 (-23.58, -3.91) | **0.006** |
| Non-metropolitan hospital | -0.20 (-0.59, 0.18) | 0.297 |  | -0.59 (-1.11, -0.07) | **0.027** |  | -46.80 (-50.51, -43.09) | **<0.001** |  | -56.48 (-60.28, -52.69) | **<0.001** |

CCI, Charlson Comorbidity index; HMO, Health Maintenance Organization; CI, confidence interval; aBeta, adjusted Beta.

^a^ Excluded patients who died in the hospital.

Significant values are shown in bold.

| **Supplemental Table S4. Multivariable analysis of the effect of frailty for outcomes.** | | | | | |
| --- | --- | --- | --- | --- | --- |
| **Outcomes** | **Frailty vs non-frailty**  **(cut at 0.36)** | | **Frailty as continuous**  **(mFI)** | |  |
|  | **aOR (95% CI)** | **p-value** | **aOR (95% CI)** | **p-value** |  |
| **In-hospital mortality ^a^** | 3.10 (0.95, 10.12) | 0.061 | 1.27 (0.95, 1.69) | 0.107 |  |
| **Complication ^b^** | 1.21 (0.64, 2.31) | 0.556 | 0.97 (0.86, 1.08) | 0.550 |  |
| **30-day readmission rate ^c^** | 1.93 (1.02, 3.68) | **0.045** | 1.15 (1.00, 1.32) | **0.046** |  |
|  | **aBeta (95% CI)** | **p-value** | **Beta (95% CI)** | **p-value** |  |
| **Length of stay, day ^d^** | 4.68 (3.59, 5.76) | **<0.001** | 0.87 (0.60, 1.14) | **<0.001** |  |
| **Total hospital cost, per 1000 USD ^e^** | 47.43 (36.18, 58.67) | **<0.001** | 12.50 (10.15, 14.85) | **<0.001** |  |

LOS, length of stay; CI, confidence interval; aOR, adjusted odds ratio; aBeta, adjusted Beta.

All outcomes excluded patients who died in the hospital: except the outcomes of in-hospital mortality and complications.

^a^ Adjusted for age, primary tumor, hospital bed size.

^b^ Adjusted for smoking status, weekend admission, hospital bed size, and hospital location/teaching status.

^c^ Adjusted for hospital bed size.

^d^ Adjusted for sex, insurance status, smoking, obesity, primary tumor, antiplatelets, systemic steroid, hospital bed size, hospital location/teaching status.

^e^ Adjusted for age, sex, smoking, primary tumor, antiplatelet drug use, anticoagulants, systemic steroids, hospital bed size, hospital location/teaching status.

| **Supplemental Table S5. The effect of frailty for outcomes (Frailty vs non-frailty) stratified by Hospital location/teaching status.** | | | | |
| --- | --- | --- | --- | --- |
| **Outcomes** | **Metropolitan teaching** | | **non-Metropolitan teaching** | |
|  | **aOR (95% CI)** | **p-value** | **aOR (95% CI)** | **p-value** |
| **In-hospital mortality ^a^** | 2.16 (0.95, 4.91) | 0.065 | 2.15 (0.31, 15.10) | 0.428 |
| **Complication ^b^** | 0.94 (0.63, 1.41) | 0.768 | 0.69 (0.26, 1.85) | 0.450 |
| **30-day readmission rate ^c^** | 1.54 (1.06, 2.23) | **0.024** | 1.05 (0.30, 3.70) | 0.943 |
|  | **Beta (95% CI)** | **p-value** | **aBeta (95% CI)** | **p-value** |
| **Length of stay, day ^d^** | 3.42 (1.92, 4.92) | **<0.001** | -2.29 (-3.93, -0.64) | **0.008** |
| **Total hospital cost, per 1000 USD ^e^** | 47.47 (26.78, 68.15) | **<0.001** | -40.52 (-67.29, -13.76) | **0.004** |

LOS, length of stay; CI, confidence interval; aOR, adjusted odds ratio; aBeta, adjusted Beta.

All outcomes excluded patients who died in the hospital: except the outcomes of in-hospital mortality and complications.

^a^ Adjusted for age, primary tumor, hospital bed size.

^b^ Adjusted for smoking status, weekend admission, and hospital bed size.

^c^ Adjusted for hospital bed size.

^d^ Adjusted for sex, insurance status, smoking, obesity, primary tumor, antiplatelets, systemic steroid, and hospital bed size.

^e^ Adjusted for age, sex, smoking, primary tumor, antiplatelet drug use, anticoagulants, systemic steroids, and hospital bed size.
